# Follow up after IMRT in oral cavity cancer: update

**DOI:** 10.1186/1748-717X-7-84

**Published:** 2012-06-11

**Authors:** Gabriela Studer, Michelle Brown, Marius Bredell, Klaus W Graetz, Gerhard Huber, Claudia Linsenmeier, Yousef Najafi, Oliver Riesterer, Tamara Rordorf, Stephan Schmid, Christoph Glanzmann

**Affiliations:** 1 Department of Radiation Oncology, University Hospital Zurich, Rämistrasse 100, 8091, Zurich, Switzerland; 2 Department of Cranio-Maxillofacial Surgery, University Hospital, Zurich, Switzerland; 3 Department of Otorhinolaryngology, Head and Neck Surgery, University Hospital, Zurich, Switzerland; 4 Department of Medical Oncology, University Hospital Zurich, Raemistrasse 100, 8091, Zurich, Switzerland; 5 Otorhinolaryngology, Klinik Bethanien, Toblerstrasse 51, 8044, Zurich, Switzerland

**Keywords:** IMRT in oral cavity cancer, Definitive IMRT for oral cavity cancer, Prognostic parameter in oral cavity cancer, Salvage treatment for recurrent oral cavity cancer, Recurrent oral cavity cancer

## Abstract

****Purpose**:**

Except for early stages (T1/2 N0), the prognosis for patients with oral cavity cancer (OCC) is known to be worse than for those with pharyngeal carcinoma. While definitive intensity modulated radiation therapy (IMRT)-chemotherapy affords loco-regional control rates (LRC) of approximately 80% in advanced pharyngeal cancer, corresponding rates are reported to be much lower for OCC. The aim of this work was to evaluate loco-regional disease control and overall survival (OAS) in a relatively large OCC patient cohort treated in the IMRT era.

****Methods and materials**:**

Between October 2002 and June 2011, 160 OCC patients were treated with curative intention IMRT at our department. 122 patients (76%) were referred with primary disease and 38 patients (24%) with a recurrent OCC at least 3 months after surgery alone. Definitive IMRT was performed in 44/160 patients (28%), whilst 116 patients underwent previous surgery. Simultaneous systemic therapy was administered in 72%.

****Results**:**

Patients with postoperative IMRT (+/−systemic therapy) with R0-1 status (n = 99) reached significantly higher LRC/OAS rates than patients following IMRT for macroscopic disease (n = 61), with 84%/80% versus 38%/33% at 3 years, respectively (p < 0.0001). This was found in patients treated for initial, as well as recurrent, disease. Less than 2% persisting grade 3/4 late effects were observed.

****Conclusions**:**

IMRT for R0-1 situations translated into a highly significant superior LRC and OAS compared to the IMRT cohort treated for macroscopic disease. Treatment was well tolerated.

## **Purpose**

Except for early stages (T1/2 N0) [1,2], the efficacy of primary radiation of oral cavity cancer (OCC) is known to be worse than for pharyngeal carcinoma. Although definitive intensity modulated radiation therapy (IMRT)-chemotherapy affords loco-regional control rates (LRC) of approximately 80% in advanced pharyngeal cancer, corresponding rates are reported to be much lower for OCC [3-5].

The impact of IMRT on the survival and local control has not been, up to now, definitively stated. The aim of this work was to evaluate the loco-regional disease control and overall survival in a relatively large OCC patient cohort treated with IMRT.

## **Methods and materials**

### **Patients**

Between October 2002 and June 2011, 160 OCC patients were treated with curative intention IMRT at our Department of Radiation Oncology, Table 1. 122 patients (76%) were referred at initial diagnosis, and 38 patients (24%) presented with a recurrence after surgery only. Definitive IMRT was performed in 44 patients (28%; mean/median primary gross tumor volume (GTV) 41 cc/29 cc (range, 0–162); mean/median nodal GTV 8.5 cc/3 cc (0–67); mean/median total GTV 49 cc/41 cc (9–162)). Details on the follow up time are listed in Table 1).

**Table 1:** Patient and disease characteristics in oral cavity cancer (OCC, N = 160)

**Parameters**	**PATIENTS**		
		**Postoperative IMRT**	**Definitive IMRT**
**N patients**		116	44
**concomittant systemic therapy**		78 (67%)	30 (68%)
**Induction chemotherapy**		2 (2%)	5 (11%)
**Gender,** male / female		69% / 31%	57% / 43%
**Age,** mean / median (range)		59 / 58 (25–90) years	66 / 64 (41–85) years
**WHO** performance status 0, 1, 2		83%, 13%, 4%	62%, 34%, 4%
**Histology:**	squamous cell carcinoma	114 (98%)	43 (98%)
	adenoid cystic carcinoma (ACC)	0	1
	Merkel cell carcinoma	2 (2%)	0
**Localization:**	T/FoM	74 (64%)	30 (68%)
	mandible/alveolar process/TR	32 (28%)	7 (16%)
	buccal mucosa (cheek)	8 (7%)	4 (9%)
	others (lip, palate)	2 (2%)	3(7%)
**T stage:**	rT0	9 (8%)	0
	rT+	17 (15%)	10 (23%)
	T1	16 (14%)	1 (2%)
	T2	35 (30%)	6 (14%)
	T3	10 (9%)	6 (14%)
	T4	29 (25%)	21 (48%)
**N stage:**	rN0	5 (4%)	2 (5%)
	rN+	15 (13%)	2 (5%)
	N0	36 (31%)	12 (27%)
	N 1a-2b	44 (38%)	13 (30%)
	N2c	10 (9%)	15 (34%)
	N3	1 (1%)	0
**Stage**	I	5 (4%)	0
	II	10 (9%)	0
	III	17 (15%)	1 (2%)
	IV	57 (49%)	33 (75%)
	nodal or primary recurrence	27 (23%)	10 (23%)
Follow up, mean/median (range)	all patients	33 / 18 (2–101) months	20 / 12 (1–87) months
	alive patients	35 / 35 (2–101) months	33 / 22 (5–87) months
	dead patients	24 / 22 (5–66) months	12 / 8 (3–27) months

Table 2 shows the analyzed population. Seventeen of 116 (15%) postoperative IMRT patients presented with macroscopic disease at the time of the IMRT planning computed tomography (CT): in two patients due to incomplete surgery (R2); in a further 15 operated patients, rapid macroscopic disease progression developed in the short interval between operation and beginning of IMRT (mean interval of 6.2 weeks, range 4–10). Ten of these 17 patients developed nodal disease only (macroscopic tumor volume 1–11 cc (mean 5.5 cc)), 2/17 had only local disease (5 and 10 cc), and 5/17 presented with loco-regional disease (13–67 cc (mean 35 cc)). Postoperatively detected macroscopic disease was histologically confirmed in patients with doubtful new lesions; in patients with clinical or radiological very obvious and suspicious lesions (newly developed, large, contrast enhancing, central necrosis), radiological as well as clinical evidence was considered sufficient for diagnosis. In all 17 postoperative patients with macroscopic disease visible in the IMRT planning CT, the ‘postoperative’ IMRT dose was increased to 70 Gy, according to the standard dose for definitive IMRT.

**Table 2:** Analyzed population, with focus on the presence of macroscopic (GTV+) versus microscopic (GTV-) disease

**160 OCC patients**	**116 postoperative IMRT (72%)**	**44 definitive IMRT (28%)**
**122 primary disease (76%)**	81: GTV−	34 (GTV+)
	7: GTV+	
**38 recurrent disease (24%)**	18: GTV-	10 (GTV+)
	10: GTV+	
**61 GTV + (%)**	**17**	**44**
**99 GTV- (%)**	99	0

The remaining 99/116 operated patients were on macroscopic evaluation disease free (resection margin free or only microscopic involved (R0-1)).

Patients referred for salvage treatment of local and/or nodal recurrence after surgery alone (n = 38) were separately analysed and outcomes compared to that in the subgroup treated for primary disease (n = 122).

In addition, the potential impact of the site of the tumor (cancer of the mobile tongue and floor of mouth versus buccal mucosa versus jaw / alveolar process / retromolar trigone versus others (lip/palate), +/− systemic therapy, gender, age, and the TN stage were assessed.

Toxicity was assessed based on RTOG/EORTC Radiation Morbidity Score. Both classifications have been considered; for simplification, grade 3 or 4 late reactions were termed 'grade 3/4' reactions. Subacute/late mucosal ulceration grade 3/4 was therefore defined as deep and/or bleeding ulcer.

Radio-osteonecrosis (RON) was scored according to a formerly described and used grading system [6]: grade 2 was defined as exposed bone with signs of infection or sequestration (corresponding to the NCI and EORTC classification of a grade 2 RON); grade 3 as RON treated with mandible resection, with satisfactory result; grade 4 as RON with persistent problems despite mandible resection; and grade 5 as death due to RON.

During the course of irradiation, all patients were clinically assessed at regular weekly intervals, and 2 weeks to 2 months after completion of treatment. Four to 6 weeks after completion of IMRT, all patients were seen regularly in our joint clinics at the Departments of Cranio-Maxillofacial Surgery and Head and Neck Surgery. Institutional standards for patient assessment included physical examination with body weight monitoring approximately every 2 months in the first year of follow-up, every 3 months in the second to third year and every 6 months in the fourth to fifth year. In addition, radiological evaluations based on CT, magnetic resonance imaging or positron emission tomography-CT was performed at 6 to 12 months intervals, or in case of suspicious findings, in parallel to histological examination.

### **IMRT: indications, technique and dosage**

General indications for postoperative radiation in operated patients were: locally advanced stages, positive surgical margins, perineural spread and involvement of 2 or more lymph nodes or extra-capsular extension. Prescribed dose ranged between 60–66 Gy in 2 Gy per fraction. The elective nodal volume was defined depending on the situation: no elective radiation in pN0-1 with sufficient number of nodes resected form the relevant nodal levels; contralateral cervical nodal irradiation (54 Gy in 1.64-1.8 Gy per fraction) for large and/or non-lateralized primaries, or >2 ipsilateral lymph node metastases, or large nodal metastasis.

Reasons for primary IMRT were patient preference (4/44) and medical and/or surgical reasons in the remaining 40 patients.

IMRT was delivered by 6 MV photon beams on a Varian linear accelerator with sliding window technique. The technical solution of choice was a 5 field arrangement (‘class solution’) for all patients. 70 Gy or 69.6 Gy in 35 and 33 fractions was administered for definitive IMRT; one patient received 74 Gy. IMRT treatment was delivered using a simultaneously integrated boost (SIB) technique in all patients; details on SIB are reported elsewhere [7]. The dose in electively irradiated regions was 54 Gy in 33 fractions (range 50–56).

The high dose planning target volume (PTV1) included the gross tumor volume (GTV) and a margin of approximately 1–1.5 cm. Elective irradiation of lymphatic regions in T3/4 or N + situations included level I,II,III and lV bilaterally of the neck and level V on the ipsilateral side. In patients with N+, the retropharyngeal nodes bilaterally were also included. On the uninvolved side of the neck, the upper field border was at the lower border of the transverse process of C1.

Patient alignment was checked before each irradiation by portal imaging; deviations of >3 mm were corrected before treatment.

Volume delineation, dose calculation and plan optimization was performed on a Varian Treatment Planning System (Eclipse®, Version 7.3.10, Varian Medical Systems, Hansen Way, Palo Alto CA, 94304–1129).

Contouring and dose distribution reviews of all cases have been performed by the first and/or the last author.

### **Chemotherapy**

Simultaneous chemotherapy was administered in 72% patients. All definitive as well as postoperative irradiated patients with no specific contra-indications underwent concomitant cisplatin chemotherapy (40 mg/m2 once weekly), or cetuximab therapy (standard dosage, 400 mg/m2 loading dose, 250 mg/m2/week afterwards; also used in operated patients with advanced disease). Seven patients with very large lesions and questionably curative options were treated with induction chemotherapy (taxotere, cisplatin, 5-fluorouracil), aiming to assess disease response with respect to a subsequent curative versus palliative treatment approach, or to ‘down stage’ tumor load prior to a surgical option. Concomitant chemotherapy was given after induction chemotherapy whenever possible/tolerated.

### **Statistics**

All statistical analyses consisted of comparing groups according to a time-to-event endpoint (survival analysis), using Kaplan-Meier curves and log-rank tests implemented in StatView® (Version 4.5). P values < 0.05 were considered as significant.

## **Results**

Fifty-three patients (53/160, 33%) experienced loco-regional treatment failure +/− distant metastases (20 patients with local failure only, 4 with nodal failure only, 7 with distant failure only, 13 with loco-regional failure, 6 with loco-regional and distant failure, 8 with local and distant and 3 with nodal and distant failure). Salvage surgery was performed in 8 of the 53 patients (15%), and was successful in 2 patients (with nodal disease only). Distant metastasis free survival was 83%. Forty-four out of the 53 loco-regional failures (83%) developed in the first 12 months after completion of therapy, 21/24 (87%) of distant failures and 45/55 (82%) of deaths occurred during the first 24 months after IMRT. 58% of all patients were alive with no evidence of disease when last seen, 9% were alive with disease, 3% had inter-currently died with no evidence of disease, and 31% had died from disease.

### **Impact of macroscopic disease**

Postoperative IMRT patients with R0-1 resection status (n = 99, 62%) reached significantly higher LRC/OAS rates than patients with macroscopic loco-regional disease (n = 61, including 44 patients treated with primary IMRT, and operated patients with residual (n = 2) or rapidly progressive disease in the interval between surgery and planned postoperative IMRT (n = 15)), p < 0.0001. Disease control rates in the definitive IMRT subgroup (n = 44) and in postoperative IMRT patients presenting with postoperative macroscopic disease (n = 17) were comparably poor, Table 3.

**Table 3:** Outcome in 160 OCC IMRT patients, analyzed according to +/− macroscopic disease prior to IMRT

**parameters**	**n (%)**	**4-y LRC**	**4-y DMFS**	**4-y OAS**
**postop IMRT (R0-1)**	99 (62)	80%	86%	79%
**postop IMRT, macroscopic disease present**	17 (11))	35%	60%	30%
**primary IMRT**	44 (27)	37%	86%	37%

Ten of the 17 patients who presented with postoperative macroscopic disease had nodal disease only; 6 of these 10 died from disease, 3 were alive with no signs of disease when last seen and one patient was alive with disease. The remaining 7 patients presenting with local +/− nodal disease all died from disease.

Seven patients were diagnosed with isolated distant metastasis only, 1–26 months (mean 10) after completion of IMRT (4% of the cohort: 3 with macroscopic loco-regional tumor treated with IMRT, 4 with R0-1 surgery prior to IMRT).

### **Impact of treatment for initial diagnosis versus salvage treatment**

**Figure 1:**
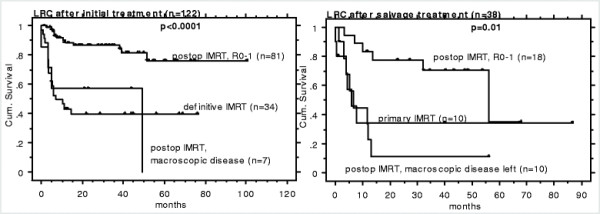
LRC following IMRT in 122 patients with initial OCC diagnosis (left) and in 38 patients with salvage treatment of OCC recurrence following surgery alone (right).

**Figure 2:**
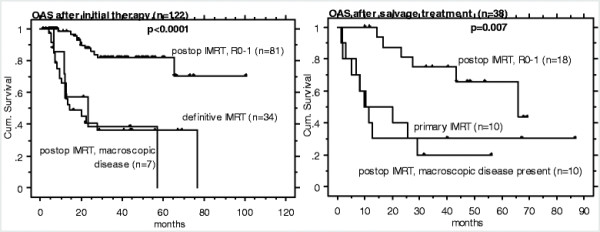
OAS following IMRT in 122 patients with initial OCC diagnosis (left) and in 38 patients with salvage treatment of OCC recurrence following surgery alone (right).

Patients referred for IMRT in the primary (n = 122) versus salvage treatment of recurrent situation (i.e. loco-regional OCC recurrence after surgery alone, n = 38, diagnosed 3–144 months (mean 30) after initial surgery only), showed comparable LRC and OAS Kaplan Meier survival curves, when analyzed according to the criterion ‘IMRT for R0-1’ versus ‘IMRT for macroscopic disease’, Figures 1 and 2, Table 2.

The small sample of patients with disease recurrence referred for salvage IMRT of macroscopic disease (n = 10/38) showed very poor outcome (LRC in 1/10, 8/10 died, 1/10 alive with disease).

Eight of 38 patients were referred for local and nodal recurrence (rT1-4 rN+), 19 for local recurrence (rT1-4 N+/− (initially N0)) and 11 for nodal recurrence only (rT0 rN+). Outcome results and comparison with the non-IMRT literature of patients treated for recurrent OCC after initial surgery only are shown in Table 4[8-11]. 

**Table 4:** Salvage treatment for recurrent OCC following initial surgery

**Author [ref]**	**Center**	**Year**	**Interval**	**N recurrences after surgery alone**	**IMRT**	**OAS rate after salvage therapy**	
						**Surgery (+/−RT)**	**RT (+/−CT)**
Schwartz et al [8]	U of Illinois, Chicago	2000	1956-1992	38 (28%)	no	~25% at 5y (n = 27)	0% at 1y (n = 11)
Koo et al [9]	Yonsei U, Seoul	2005	1991-2003	36/127 (28%)	no	~25% at 5y (n = 13)	0% at 2y (n = 10)
Liu et al [10]	U of Taiwan	2007	1995-2003	224 (na)	no	34% at 5y (n = 326)∗	21% at 2y (n = 75)∗
Kokemueller et al [11]	U of Hannover	2011	1980-2009	115/341 (37%)	no	~25% at 5y (n = na)	10% at 4y (n = na)
own group	U hospital of Zurich	2012	2002-2011	38 (na)	yes	55% at 5y (n = 28)	30% at 5y (n = 10)
						75% at 5y (n = 18)∗∗	25% at 5y (n = 20)∗∗

∗: outcome of recurrent OCC following surgery alone (n = 224) and surgery + RT/CT (n = 177).

∗∗: results when analyzed according to ‘salvage IMRT for R0-1 situations’ versus ‘salvage IMRT for macroscopic disease’. U: University. na: not available.

Eighteen of the 38 (47%) patients with recurrence after surgery could undergo R0-1 salvage surgery (Figure 1 and 2, right). This subgroup showed comparable outcome as patients with R0-1 surgery followed by IMRT at initial diagnosis (n = 81, Figure 1 and 2, left).

### **Impact of other parameters**

Operated T1 patients attained 100% LRC at 4 years (n = 17), while operated stage T2-4 patients reached approximately 70–80% LRC and definitively irradiated T1-4 patients 30–40% LRC (n cT1 = 1). WHO performance status showed to be a significant outcome predictor in the small recurrence subgroup (n = 38, p < 0.005), while no significant difference was found for the larger subgroup referred for initial disease (p = 0.06). The difference in the performance status between operated and definitively irradiated patients (PS 1–2 17% vs 38%) was not found to translate in significantly different LRC or OAS. Systemic therapy (none versus any) and different systemic treatment schedules (cisplatin or cetuximab, number of cycles, concomitant +/− induction) did not show significant outcome differences between the small subgroups.

The impact of the tumor site was assessed according to the sites as listed in Table 1. No differences were seen between the small subgroups when analyzed for the entire group as well as for patients treated with IMRT for R0-1 versus macroscopic disease. Gender and age also demonstrated no impact on outcome.

### **Grade 3/4 late term treatment effects**

Treatment tolerance following IMRT with high boost doses of 60–65 Gy (n = 22), 66 Gy (n = 68), 68 Gy (n = 3), 69.6 Gy or 70 Gy (n = 66) or even 74 Gy (n = 1) was high, considering that additional systemic therapy was delivered in 72% of the population. One patient developed a grade 2–3 RON of the mandible after IMRT, which was successfully treated by limited surgery (partial decortication) after 7 months of conservative treatment. One patient developed a RON following insertion of dental implants. Two patients remained gastric feeding tube dependent (one of them after extensive floor of mouth tumor surgery). There were no patients with persisting relevant dysphagia or severe grade 3/4 xerostomia. Four patients developed sub-acute mucosal ulcers grade 4 in the area of former primary tumor and IMRT boost dose, which healed after several months (3, 5, 7, and 11 months after the completion of IMRT).

## **Discussion**

The purpose of the current study was to evaluate the loco-regional disease control and overall survival in our large OCC patient cohort treated with IMRT with a longer follow up than other contemporary published series. The first 57 previously analyzed OCC patients treated with IMRT at our institution [5] (see also Table 4) have been updated and are included in this recent evaluation. The main limitation of this study is its retrospective character, and the therefore (expectedly) unbalanced subgroups (definitive vs postoperative IMRT, patients referred for primary disease vs for recurrence, Table 2). The subgroups are also expectedly unbalanced with respect to the performance status (PS 1–2 38% vs 17% in definitively irradiated vs operated patients) and the primary stage (T3-4 in 62% versus only 34% in operated patients).

We found the presence of macroscopic disease to be a highly predictive unfavourable outcome parameter. Initial treatment with primary IMRT (without surgery) resulted in inferior outcome compared to primary surgery followed by IMRT. Primary IMRT in OCC also achieved inferior control rates compared to primary IMRT in all other head and neck sites; unfavourable outcome in patients irradiated for macroscopic tumor seems characteristic for OCC, contrary to primary IMRT for pharyngeal squamous cell carcinoma, which translates to very satisfactory loco-regional control rates of ~80% (+/− simultaneous systemic therapy) [3,4,10]. The reason for this difference remains unclear.

Our OCC patients receiving definitive radiation in the analyzed cohort represent an unfavourable selection – even the 7 patients with early primaries (cT1-2) all had advanced nodal disease (N2c (n = 6), N2a (n = 1)). Nevertheless, comparable loco-regional disease at other head and neck sites achieves much higher control rates. Contrary to these advanced stages, excellent results can be reached in cT1N0 and limited cT2N0 by interstitial brachytherapy [2].

Operated OCC patients with early recurrence developing in the short interval between surgery und postoperative IMRT certainly also represent an unfavourable group with very aggressive tumor features – the potential benefit of a re-operation in such situations remains speculative. This subgroup showed a similarly poor outcome comparable to the definitive IMRT subgroup.

As the other published OCC results are also based on retrospective case series (Table 4 and 5) there is no definitive randomized evidence so far of the value of postoperative radiation.

**Table 5:** Selected publications on disease control rates following postoperative (n > 300) and definitive IMRT (n = 63) in OCC

**Author [ref]**	**Center**	**Year**	**Interval**	**N**	**postop IMRT**	**CT**	**T3/4**	**N>/=2**	**lll/lV**	**LRC**		**D M F S**		**O A S**	
										**Postop**	**Definitive**	**Postop**	**Definitive**	**Postop**	**Definitive**
Eisbruch et al [3]	U of MI	2004	1997-2002	27	most postop	na	na	na	na	59% (3y)	na	na	na	na	na
Yao et al [4]	U of Iowa	2007	2001-05	55	49 (89%)	11%	56%	36%	91%	85% (3y)	na	89% (3y)	na	68% (3y)	na
Studer et al [5]∗	U of Zurich	2007	2002-07	58	28 (48%)	78%	69%	28%	62%	91% (2y)	43% (2y)	95% (2y)	85% (2y)	83% (2y)	30% (2y), n = 30
Gomez et al [12]	MSKCC	2009	2000-06	35	35 (100%)	29%	40%	38%	80%	77% (3y)	none	85% (3y)	na	74% (3y)	none
Chen WC et al [13]	Chiayi, Taiwan	2009	2002-05	22	22 (100%)	na	na	7 (32%)	100%	64% (3y)	none	na	na	67% (3y)	none
Collan et al [14]	U of Helsinki	2011	2001-2007	40	40 (100%)	38%	na	na	na	na	none	na	na	75% (3y)	none
Sher D et al [15]	DFCI	2011	2004-09	42	30 (71%)	~76%	45%	30%	64%	91% (2y)	64% (2y)	94% (2y)	83% (2y)	85% (2y)	63% (2y), n = 12
Daly M et al [16]	Stanford UMC	2011	2002-09	37	30 (81%)	68%	54%	46%	57%	53% (3y)	60% (3y)	81% (3y)	71% (3y)	60% (3y)	57% (3y), n = 7
own cohort	U of Zurich	2012	2002-2011	160	99 (62%) ∗∗	72%	40%	77%	68%	84% (4y)	40% (4y)	90% (4y)	85% (4y)	81% (4y)	38% (4y), n = 44

(∗): included in the recent analysis.

(∗∗): included all patients with R0-1 status (excluded 17 operated patients with macroscopic disease after surgery.

U: University Hospital.

MSKCC: Memorial Sloan-Kettering Cancer Center.

DFCI: Dana-Farber Cancer institute.

UMC: University Medical Center.

CT: Chemotherapy.

LRC: loco-regional control.

DMFS: distant metastasis free survival.

OAS: overall survival.

Outcome data on OCC treated with IMRT remain scant. Table 5 shows published outcome results for IMRT in OCC [3-5,12-16]. To our knowledge, only two other reports are available on definitive IMRT patients to date: Sher et al [15] observed LRC/OAS rates of 64%/63% at 2 years in 12 patients, Daly et al [16] reported on 7 definitive IMRT patients, with corresponding rates of 60%/56% at 3 years, respectively. Our LRC/OAS rates in 44 definitively irradiated patients were ~40% each at 4 years.

A comparison with the literature of our 38 patients referred for (definitive or postoperative) salvage IMRT for recurrence after surgery is listed in Table 4[8-11]: the comparison is based on non-IMRT literature, as no data is available regarding radiation of recurrent disease from the IMRT era. There is, so far, no evidence for superiority of IMRT compared to non-IMRT techniques with respect to OCC disease control [5,13]. Our small sample of patients with recurrent macroscopic disease referred for IMRT (n = 10/38) showed very poor outcome (LRC in 1/10, 8/10 died, Figures 1, 2), as also reported by Koo et al [9] and Schwartz et al [8]. In summary, these studies reported OAS of about 25–35% at 5 years following salvage surgery (+/−radiation) of loco-regionally recurrent disease initially treated with surgery only, Table 4[8-11]. The OAS rate following definitive salvage radiotherapy in those patients was as low as 0- ~ 20%. Our IMRT results are somewhat better; when analysed according to the criterion IMRT for ‘R0-1’ versus ‘macroscopic disease’, satisfactory OAS of 75% at 5 years were attained for 18 patients undergoing radical salvage surgery followed by IMRT.

Poor OAS of approximately 20–30% at 2 years after salvage therapy is also reported for nodal recurrence following initial surgery +/− radiation (+/−chemotherapy) [17,18].

The IMRT tolerance in OCC patients was high, confirming earlier results regarding RON of the mandible and occurrence and nature of sub-acute mucosal ulcers in the IMRT boost dose area [6,7,19-21]. However, no specific investigations (e.g. quantitative measurement of salivary flow or quality, swallowing studies) have been performed to assess xerostomia and dysphagia but clinical follow up examinations (as mentioned in ‘methods’) and routine chart reviews on patients’ complaints were undertaken. The fact that only 2 patients remained feeding tube dependent may serve as a surrogate for sufficient swallowing capacity < grade 3 toxicity.

## **Conclusions**

IMRT for postoperative R0-1 situations translated into a significant superior LRC and OAS compared to the IMRT cohort treated for macroscopic disease

Patients with recurrent disease (following surgery alone) showed similar outcome as patients irradiated for the initial situation, when analyses were performed according to the criteria ‘IMRT for R0-1’ versus ‘IMRT for macroscopic disease’

Persistent grade 3/4 late effects following IMRT (+/- systemic therapy) developed in <2% of our cohort

These updated findings corroborate our earlier recommendation in favour of a multi-modality approach with surgery followed by postoperative IMRT(-chemotherapy) in advanced OCC.

## **Competing interests**

The authors declare that they have no competing interests.

## **Authors' contributions**

GS, MB1 and CG designed and drafted the manuscript. MB1 and MB2 participated in its design and helped to draft the manuscript. All authors were responsible or substantially involved in patients’ specific treatments and clinical follow up, and generated the presented follow up data. All authors read and approved the final manuscript.
